# Pediatric Rheumatological Diseases in a Tertiary Care Hospital of Central India: A Retrospective Clinico-Epidemiological Profile

**DOI:** 10.7759/cureus.53327

**Published:** 2024-01-31

**Authors:** Tushar B Jagzape, Priyanka Pandey, Turaka Silpa, Shirisha Pinky

**Affiliations:** 1 Department of Pediatrics, All India Institute of Medical Sciences, Raipur, Raipur, IND

**Keywords:** epidemiological profile, clinical profile, retrospective study, rheumatological disorders, kawasaki disease (kd), systemic lupus erythematosus (sle), juvenile idiopathic arthritis (jia)

## Abstract

Introduction*: *Infectious diseases account for the major health problem in developing countries like India. Though non-infectious diseases like rheumatological disorders are not very common, the burden of these disorders as a group is high in society due to the huge population size. The rheumatological disorders have varied presentations which may mimic other infectious pathologies leading to a significant time lag in the diagnosis. There is inadequate data on the exact burden of these diseases. The spectrum of rheumatological disorders in developing countries is different as compared to the Western world. Hence this study was carried out with the aim of studying the clinical, epidemiological, and laboratory profile of rheumatological disorders in the pediatric age group in a tertiary care hospital.

Methods*:* It was a retrospective study. Data of patients admitted with the diagnosis of rheumatological disorder in the age group of one month to 15 years during the period from June 2018 to December 2022 were reviewed.

Results*:* A total of 35 patients were identified with 20 being female. The mean age of the patients was 8.42± 3.95 years. The most common disease was juvenile idiopathic arthritis (JIA)- 10(28.57%) with an equal proportion of polyarticular JIA and systemic-onset JIA, followed by systemic lupus erythematosus (SLE) nine (25.71%) and Kawasaki Disease (KD)- eight (22.85%). The commonest presenting complaint was fever followed by a rash, whereas the most common findings were pallor and rash. Anemia was present in 25 (71.42%). C-reactive protein (CRP) and erythrocyte sedimentation rate (ESR) were high in 20 (57.14%) and 22 (62.85%), respectively. Antinuclear antibodies (ANA) were positive in 10 (28.57%) and rheumatoid factor (RA) factor in only one (2.85%) case.

Conclusions*:* The most common rheumatological disorder identified was JIA. Fever and rash were the common presenting complaints. Pallor was the commonest sign whereas anemia was the commonest hematological abnormality.

## Introduction

Though infectious diseases are a major burden in the pediatric population, rheumatic disorders are also not uncommon. They result in significant morbidity and mortality [1]. Different regions across the world have reported different prevalences of various rheumatic diseases in children. The reported prevalence of juvenile idiopathic arthritis (JIA) is 0.07 to 10/1000 children and 0.4 to 0.6/100000 of systemic lupus erythematosus (SLE) [2,3]. About 10%-20% of the rheumatological disorders have their initial presentation in the pediatric age groups. Significant disability affecting economic productivity and disability-adjusted life years are the consequences of delayed diagnosis and appropriate interventions [4]. There is inadequate data for the exact burden of rheumatic diseases in children from India, but extrapolating from the above population data, there could be not less than 1.3 million children with JIA in India and approximately two lakh children with SLE [5]. World Health Organization (WHO) and the European League Against Rheumatism (EULAR) ran a decade-long bone and joint campaign (2000-2010) with the aim of creating global awareness about musculoskeletal conditions considering the high global burden due to long-term disability, pain, and poor quality of life due to musculoskeletal conditions [6,7].

Since rheumatological disorders may present with non-specific features like fatigue, growth failure, back pains, or deterioration in school performance [8]. When compared to Western nations, there is a difference in the clinical spectrum of many rheumatological disorders in India [9]. It is therefore necessary for the clinician to be cognizant of a varied range of conditions that can cause such symptoms in childhood and adolescence and its assessment to arrive at the correct diagnosis and plan further management.

Hence, this retrospective study was carried out with the aim of studying the clinical epidemiological and laboratory profile of rheumatological disorders in the pediatric age group in a tertiary care hospital in central India. The objectives of the study were to study the clinical features of various rheumatological diseases admitted in the Department of Pediatrics at All India Institute of Medical Sciences (AIIMS), Raipur, Chhattisgarh, India and to characterize the hematological and biochemical profile of these patients. The extracted data might help in creating awareness among the treating clinicians and helping in early identification, diagnosis, and referral to experts with organized healthcare for such children.

## Materials and methods

This study was conducted in the Department of Pediatrics of AIIMS, Raipur. This is a tertiary care teaching hospital located in the central East part of India and caters to the health needs of the population of the state of Chhattisgarh and surrounding states. A large percentage of the population in this state belongs to the tribal communities.

It was a retrospective study. Medical records of the patients admitted to the Department of Pediatrics, AIIMS, Raipur between the period from June 1, 2018 to December 31, 2022 were reviewed. Pediatric patients up to the age of 15 years are admitted in the pediatric department, hence the study age group was restricted from one month to 15 years. The patients with the diagnosis of JIA, SLE, enthesitis-related arthritis, juvenile dermatomyositis, Kawasaki Disease (KD), and other vasculitis syndromes (Henoch-Schönlein purpura), Takayasu arteritis, polyarteritis nodosa, antineutrophilic cytoplasmic antibody-associated vasculitis, and other connective tissue disorders were selected. The diagnosis at the time of discharge was used to identify the patients with these diseases. The diagnosis and classification of JIA were done as per the International League of Associations for Rheumatology (ILAR) classifications of childhood chronic arthritis. The Systemic Lupus International Collaborating Clinics (SLICC) classification criteria were used for the diagnosis of SLE. Other diseases were diagnosed based on the clinical and laboratory features diagnostic of the illness. The patients in whom the diagnosis was not confirmed and in whom a lot of data were missing were excluded. From these records, the demographic details were entered into a Performa. The history was reviewed, and the presenting complaints were identified. The physical examination findings were used to pick up the signs. The laboratory data was also screened and the required parameters were recorded in the Performa. Once all the data were collected it was entered into a Microsoft Excel sheet and data analysis was done. For continuous data like age mean was calculated. For discreet data like symptoms and clinical signs, frequency was calculated and expressed as a percentage. Inferential statistics was not used.

The proposal was duly approved by the Institute Ethics Committee (IEC) of AIIMS, Raipur, Chhattisgarh vid letter no. 2086/IEC-AIIMSRPR/2021 dated 27/12/2021. Since it was a retrospective record review study consent waiver was granted by the IEC.

## Results

In this retrospective study, we could retrieve records of 35 patients. The ratio of male to female was 1:1.33 Maximum patients were in the age group of 10-15 years (Table 1).

**Table 1:** Demography of the patients (N=35)

Age group (Year)	Male (%)	Female (%)	Total (%)
0-5	6 (17.14)	1 (2.85)	7(20)
5-10	2 (5.71)	7 (20)	9(25.71)
10-15	7 (20)	12 (34.28)	19(54.28)
Total	15 (42.85)	20 (57.14)	35 (100)

The most common presenting symptom was chronic fever (intermittent or relapsing) seen in 29(82.85%), followed by rash in 21 (60%), joint pain in 12 (34.28%), and joint swelling in eight (22.85%). Edema all over the body was the presenting complaint in three patients with lupus nephritis. On clinical examination, the common findings in decreasing frequency were pallor 27 (77.14%), rash 21(60%), hepatomegaly 11 (31.42%), joint swelling eight (22.85%), splenomegaly eight (22.85%), and lymphadenopathy five (14.28%). The common symptoms and signs are depicted in Table 2.

**Table 2:** Common symptoms and signs

Sn	Symptoms (%)	N (%)	Sign	N (%)
1	Fever	29 (82.85)	Pallor	27(77.14)
2	Rash	21 (60)	Rash	21(60)
3	Joint pain	12 (34.28)	Lymphadenopathy	5(14.28)
4	Joint swelling	8 (22.85)	Edema feet	4(11.42)
5	Muscle pain	1 (2.8)	Hepatomegaly	11(3.14)
6	Back pain	1 (2.8)	Splenomegaly	8(22.85)
7	Fatigue/malaise	6 (17.14)	Joint swelling	8(22.85)
8	Paleness	5 (14.28)	Joint deformity	3(8.57)
9	Oral ulcer	4 (11.42)	Ascites	4(11.42)
10	Swelling all over the body	3 (8.57)	Pleural effusion	3(8.57)
11	Breathlessness	3 (8.57)	Pericardial effusion	2(5.71)
12	Chest pain	3 (8.57)	Anasarca	3(8.57)
13	Cough	3 (8.57)	Hemiplegia	1(2.8)
14	Distension of abdomen	2 (5.71)	Muscle tenderness	1(2.8)
15	Headache	2 (5.71)	Rheumatoid nodule	0
16	Convulsion	1 (2.8)	Uveitis	0
17	Weakness of one side of the body	1 (2.8)		

**Figure 1:**
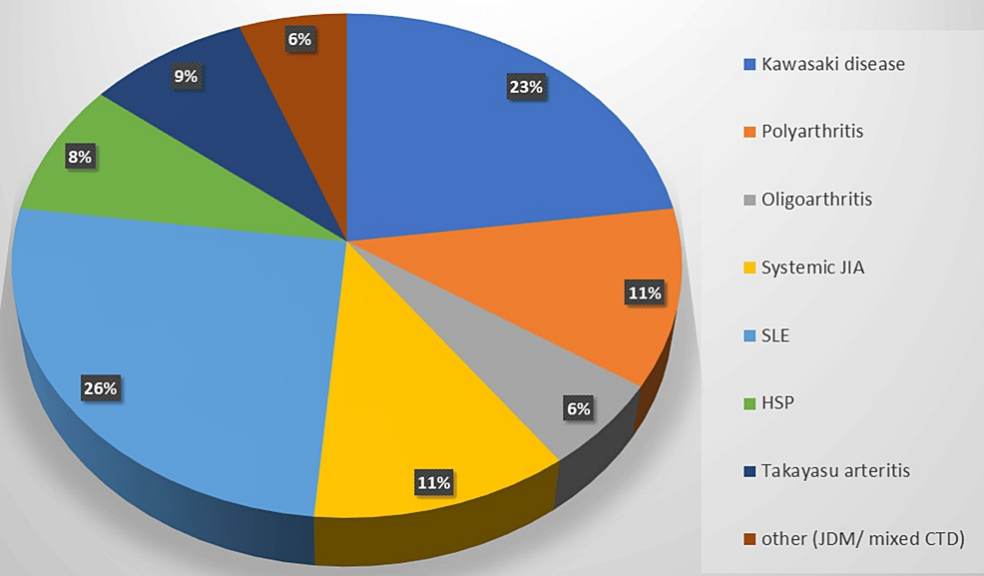
Distribution of various rheumatological disorders in this study. JIA: juvenile idiopathic arthritis; SLE: systemic lupus erythematosus; HSP: Henoch-Schönlein purpura; JDM: juvenile dermatomyositis; CTD: connective tissue disorder

Figure 1 represents the distribution of various rheumatological diseases observed in our study during the study period.

The most common diagnosis was JIA [10], followed by SLE and Kawasaki Disease. In JIA, both systemic JIA and polyarthritic JIA constituted 40% each.

The laboratory parameters of the study population are given in Table 3.

**Table 3:** Abnormal laboratory parameters (N=35) *Minimum and maximum counts per cubic mm

S. no	Parameter	Abnormal N (%)
1	Hemoglobin (Hb)	25 (71.42)
2	Total leukocyte count (TLC)	16 (45.71)*(2800-34,300)
3	Platelet count (PLT)	18(51.42)*(1,04,000-9,92,000)
4	Liver function test (LFT)	3 (8.57)
5	Renal function test (RFT)	7 (20)
6	C-reactive protein (CRP)	20 (57.14)
7	Erythrocyte sedimentation rate (ESR)	22 (62.85)
8	Creatinine phosphokinase (CPK)	2 (5.71)
9	Lactate dehydrogenase (LDH)	11 (31.42)
10	Urine- routine examination and microscopy	17 (48.57)
11	Antinuclear antibody (ANA)	10 (28.57)
12	Human leukocyte antigen (HLA) B-27	2 (5.71)
13	Rheumatoid arthritis (RA)- factor	1 (2.85)

The most common abnormality was low hemoglobin in 25 (71.42%) as per age and gender. Inflammatory markers like ESR (Range 8-130 at the end of one hour) and CRP (Range 1 to 331 mg/dl) were increased in 62.85% and 57.14% of the cases, respectively. ANA was positive in 10 (28.57%) and RA factor was positive in only one (2.85%). Abnormalities in platelet count and total leukocyte count were also common.

The summary of common findings is summarized in Table 4.

**Table 4:** Summary of important findings of the study HSP: Henoch-Schonlein purpura; TA: Takayasu arteritis; JDM: juvenile dermatomyositis; MCD: mixed connective tissue disorder; KFT: kidney function test, LFT: liver function test; KD: Kawasaki Disease

Parameters	JIA=10(28.57%)	SLE= 9 (25.71%)	KD=8 (22.85%)	Others=8 (22.85%) (HSP-3/TA-3/JDM-1, MCD -1)	Total=35
Mean age	8.5 (6.02-11.07)	10.7 (8.6-12.8)	4.43 (1.59-7.27)	9.6 (Median-10.5)	
Commonest presenting complaint	Joint pain, swelling	Fever, rash	Fever, rash	Rash, headache	
Commonest findings	Pallor, joint swelling	Pallor, rash	Rash, hepatomegaly	Rash, high blood pressure	
Hb	7 (70)	8 (88.88)	6 (75)	4 (50)	25(71.42%)
TLC	6 (60)	3 (33.33)	4 (50)	3 (37.5)	16(45.71%)
PLT	3 (30)	2 (22.22)	6 (75)	7 (87.5)	18(51.42%)
ESR	8 (80)	8 (88.88)	5 (62.5)	1 (12.5)	22(62.85%)
CRP	7 (70)	4 (44.44)	4 (50)	5 (62.5)	20(57.14%)
Deranged LFT	1 (10)	0	2 (25)	0	3(8.57%)
Deranged KFT	2 (20)	4 (44.44)	0	1 (12.5)	7(20%)
ANA	1 (10)	8 (88.88)	0	1 (12.5)	10(28.57%)
RA factor	1 (10)	0	0	0	1(2.85%)
HLA-B-27	1 (10)	1 (11.11)	0	0	2(5.71%)

## Discussion

During the study period, a total of 35 patients were diagnosed with one of the many rheumatological diseases. Out of these, 15 (42.85%) were males with the male to female ratio of the study population being 1: 1.33. A maximum number of patients were between 10 and 15 years of age (Table 1).

In a study done by Patra and Kumar, out of 60 children enrolled, the male to female ratio was 1.6:1 comprising 37 males and 23 females. Though the mean age of symptom onset of these patients was 9.1±3.6 years, there was a delay of approximately two years before they were enrolled in the rheumatology clinic [10]. Hegde et al. in their study of the clinical profile of JIA from a tertiary care hospital enrolled 56 children with 38 (67.85%) being boys and 18 (32.15%) were girls, with a male to female ratio of 2.1:1 [11]. A study on the profile of JIA from Chandigarh had a male to female ratio of 1.8:1. Eleven (14.8%) were less than five years of age [12].

This contrasts with our study where we had more female patients, although the mean age in our study was 8.42+/- 3.95 years which is similar to the above study. Our findings are in concordance with Furia et al., who in their study in a tertiary care hospital in Africa, had a study population of 52 children. They had a greater number of female subjects 32 (61.5%) with the mean age of study participants being 9.5 ± 4.3 years with 12 (40.4%) being above 10 years [1].

The most common rheumatological disorder we encountered was JIA, with 10 patients accounting for approximately (28.57%) of all cases. We had almost a similar number of cases of SLE with nine (25.71%) followed by KD eight (22.85%). The remaining cases were of HSP and Takayasu arteritis three each and one case each of juvenile dermatomyositis and mixed connective tissue disorder. Out of a total of 10 patients of JIA, polyarthritic and systemic JIA was 40% each, and oligoarthritic JIA was seen in 20% of cases. Seventy percent of the patients were male and 30% were female. In SLE, seven out of nine patients were female. All eight patients with KD were male. In both HSP and Takayasu arteritis, two patients were female and one was male. The distribution of various cases in our study is depicted with the help of the pie chart in Figure 1.

Patra and Kumar, in their cohort, also had JIA as the commonest disease 48 out of 60 accounting for almost 80% of all rheumatological disorders, which is very high as compared to our cohort. The distribution of polyarthritic and oligoarthritic JIA was 33% and 25%, which is similar to our study [10]. Systemic-onset JIA was relatively more common in our study. Singh et al. in their study had a higher percentage of oligoarthritic JIA - 35 (47.3%), followed by polyarthritis - 28 (37.8%), and only 11 (14.9%) were systemic JIA [12]. In one of the largest cohort studies conducted in India by Kunjir et al. on the profile of Indian patients with juvenile-onset chronic inflammatory joint disease, the largest group consisted of enthesitis-related arthritis 36% followed by polyarticular JIA 29%. Only 8% of patients had systemic-onset disease [13].

In another recent study published by Hegde et al., enthesitis-related arthritis was the commonest type with 20 out of 56 (35.7%), followed by 16 (28.5%) polyarthritis, and 15 (26.8%) as systemic onset [11]. A study by Furia et al. in Tanzania also reported JIA as the commonest diagnosis 28 (53.8%) followed by SLE eight (15.4%), mixed connective tissue disease and juvenile dermatomyositis both with four (7.7%) patients [1]. Studies from Eastern and Northern India have also reported polyarthritic JIA as the most common subtype in Indian children in contrast to Western countries where the oligoarthritic subtype is more common [14-16].

The most common presenting symptom was fever (chronic and relapsing) seen in 29 (82.85%) followed by rash (SLE, HSP, JDM) in 21 (60%), joint pains in 12 (34.28), joint swelling in eight (22.85%) patients. Table 2 shows the frequency of various common presenting symptoms in our study. All patients with SLE had a typical rash, whereas two out of three patients with HSP had a rash at the time of presentation. One patient’s rash had resolved when she presented to us with features of nephritis. Oral ulcers and CNS symptoms were not very commonly observed in our cohort. In the study by Furia et al., the commonest presenting complaint was joint pain 44 (84.6%). Other common complaints were joint swelling in 34 (65.4%), fever in 24 (46.2%), and skin rash in 21 (40.4%) [1]. These findings are quite different from our study. The reason could be more patients in our cohort had systemic-onset JIA and SLE which mostly present with pyrexia of unknown origin. The ethnic and environmental differences could also be responsible for the same.

On clinical examination pallor was the most common finding in 27 out of 35, that is, 77.14%, followed by rash in 21 (60%), hepatomegaly in 11 (31.42%), joint swelling and splenomegaly in eight (22.85%). As per Seth et al., when they evaluated patients with JIA, only rash was seen in 5% of cases and the percentage of hepatomegaly was very high 51%. Lymphadenopathy was also quite common 25% [14]. As per Hegde et al., fever, rash, lymphadenopathy, and hepatosplenomegaly were seen almost in all patients with systemic-onset JIA [11]. We did not find any child with a rheumatoid nodule. As can be derived from the clinical signs mentioned in Table 2, the findings are similar to those reported by Hegde et al. and Suni et al. [11,17]. Uveitis was also not seen in any of our patient. Many Asian studies including a study done by Yu et al. in Taiwan, which was a nationwide study, have reported that uveitis is not very commonly seen in the Asian population [18].

While considering the laboratory profile, a high percentage of patients had anemia as per the WHO references, that is, 25 out of 35 (71.42%). Abnormality in the TLC was also common, 16 (45.71%). Out of these, 15 cases had elevated total leukocyte count. ANA was positive in 10 (28.57%). RA factor was positive in one (2.85%). HLA B27 was positive in two (5.71%). The overall positivity rate is low. Derangement of liver function was less as compared to the kidney functions which was deranged in seven (20%) patients. LFT was deranged in three (8.57%) patients two of these were of KD and six of the seven patients with deranged KFT were cases of lupus. Singh et al. reported anemia in 26 out of 74 patients, that is, 35.13%, which is quite less than our study. The reason could be this is an old study and they have taken a cut of 10 g/dl for diagnosis of anemia irrespective of the age [12]. In the same study, they reported high ESR in 65 (87.83%) and high TLC in 31 (41.89%) cases. Seth et al. reported a high percentage of anemia in systemic JIA. RA factor positivity was 15% in polyarthritic JIA, whereas antinuclear antibody was detected in only three out of the 66 patients in whom the test was carried out [12]. In the study by Furia et al., anemia was reported in 35 (67.3% ) of the patients with hemoglobin ranging from 3.1 to 15.4 g/dl. ESR was elevated in 34 (65.38%) cases and CRP was elevated in 32 (61.5%) patients. In this study, ANA was done in 16 patients, nine of which came positive, and dsDNA was done in nine patients out of which five were positive [1]. These results are similar to our findings. Other studies also reported elevated inflammatory markers like ESR, CRP, and serum ferritin in systemic JIA [11,19]. Similar to our study, most of the Indian studies have reported a low ANA positivity rate [9,10,11].

The limitation of the study was that being a retrospective study, few of the information related to demography was missing. A few cases may have been missed due to incorrect coding.

## Conclusions

In this retrospective study involving 35 patients, male to female ratio was 1:1.33 with a mean age of 8.42 years. The most common rheumatological disorder was JIA with systemic JIA and polyarthritic JIA having equal number of patients followed by SLE. The distribution of the cases is different as compared to studies reported from other parts of India. The common presenting complaints were chronic fever and rash. Pallor was the most common finding on examination followed by a rash. ANA and RA factor positivity rates were very less (28.57% and 2.85%, respectively) which is similar to other Indian studies.
